# Supplementary material for: Maternal transmission of bacterial microbiota during embryonic development in a viviparous lizard
Source: Microbiol Spectr. 2023 Oct 17;11(6):e01780-23. doi: 10.1128/spectrum.01780-23 (PMC10714757; doi:10.1128/spectrum.01780-23)
Supplement: Supplemental material — Tables S1 and S2; Fig. S1 and S2. [file spectrum.01780-23-s0001.docx]

**SUPLEMENTARY MATERIAL**

**SUPPLEMENTAL TABLES**

**Table S1** Results of quality filtering of 16S rRNA sequencing data sets of maternal and embryonic samples of female gravid *Sceloporus grammicus* (Wiegmann, 1828).

| Sample | Location | *N* | Raw | Filtered | Denoised | Merged | Non-chimeric |
| --- | --- | --- | --- | --- | --- | --- | --- |
| **Mother** | Mouth | 8 | 9399 ± 3329 | 8582 ± 3065 | 8542 ± 3032 | 8387 ± 2936 | 8262 ± 2794 |
|  | Cloaca | 8 | 16597 ± 9056 | 15328 ± 8328 | 15282 ± 8293 | 15082 ± 8143 | 14533 ± 7735 |
|  | Small intestine | 5 | 3379 ± 4996 | 2700 ± 4066 | 2511 ± 3986 | 2355 ± 3861 | 2272 ± 3765 |
|  | Aseptic ventral skin | 8 | 8907 ± 4828 | 8118 ± 4395 | 8089 ± 4389 | 7973 ± 4344 | 7934 ± 4324 |
|  | **Mean** |  | **10211 ± 7508** | **9301 ± 6926** | **9236 ± 6924** | **9080 ± 6830** | **8869 ± 6554** |
| **Embryo** | Amniotic fluid | 9 | 13599 ± 3558 | 12279 ± 3243 | 12230 ± 3247 | 12078 ± 3210 | 11986 ± 3188 |
|  | Embryonic gastrointestinal tract | 17 | 20221 ± 19500 | 18084 ± 17686 | 17986 ± 17662 | 17796 ± 17451 | 17545 ± 17100 |
|  | Membrane | 6 | 12753 ± 3182 | 11230 ± 2677 | 11067 ± 2631 | 10973 ± 2605 | 10887 ± 2599 |
|  | Yolk | 4 | 10521 ± 2367 | 9621 ± 2196 | 9591 ± 2193 | 9531 ± 2204 | 9490 ± 2199 |
|  | **Mean** |  | **16243 ± 14140** | **14550 ± 12798** | **14461 ± 12779** | **14311 ± 12627** | **14151 ± 12373** |
| Total | **Mean** |  | **13552 ± 12036** | **12208 ± 10905** | **12130 ± 10889** | **11977 ± 10765** | **11794 ± 10529** |

**Table S2** Hill numbers of the bacterial communities maternal and embryonic samples of female gravid *Sceloporus grammicus* (Wiegmann, 1828).

| **Sample** | **Effective number of amplicon sequence variants** | | |
| --- | --- | --- | --- |
|  | ***q* = 0** | ***q* = 1** | ***q* = 2** |
|  | **mean** | **mean** | **mean** |
| **Mother** | 53.7 | 11.6 | 6.7 |
| **Embryo** | 26.1 | 4.8 | 3.3 |
| ***F* value^a^** | 9.4 | 5.6 | 3.8 |
| ***p* value** | **0.003** | **0.020** | 0.054 |
| ***p* value**  **(perm) ^b^** | **0.001** | **0.007** | **0.034** |

^a,b^ Linear mixed effects model with 1000 Monte-Carlo permutations and maternal and embryo identities as random factors was used to test the significant differences.

**SUPPLEMENTAL FIGURES**


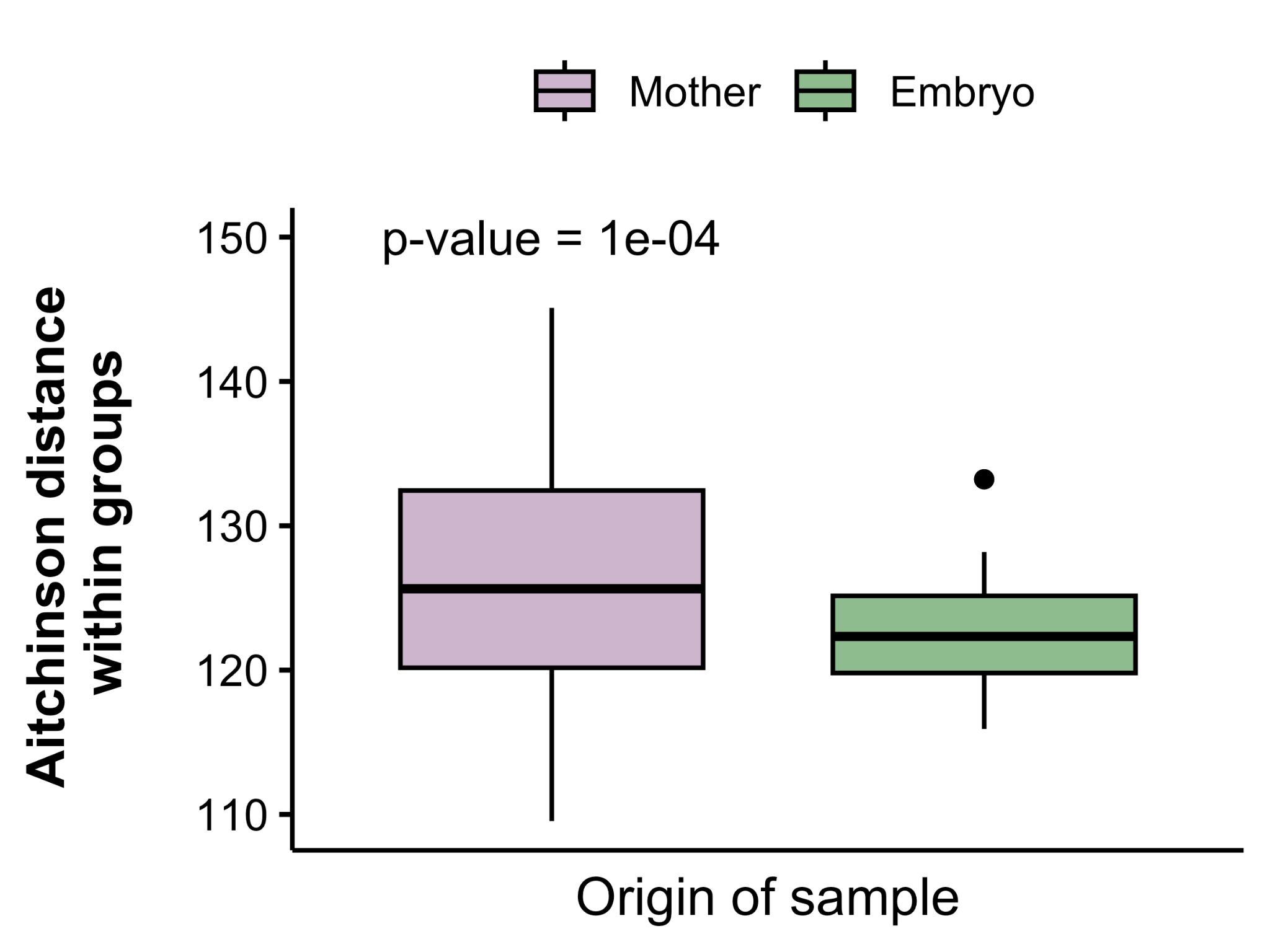


**FIG S1** Aitchinson distances of the bacterial communities of maternal and embryonic samples of female gravid *Sceloporus grammicus* (Wiegmann, 1828). Significant difference was calculated with the Fisher-Pitman permutation test with 10,000 Monte-Carlo permutations. Pairwise comparisons occurring between the same individual were selected.


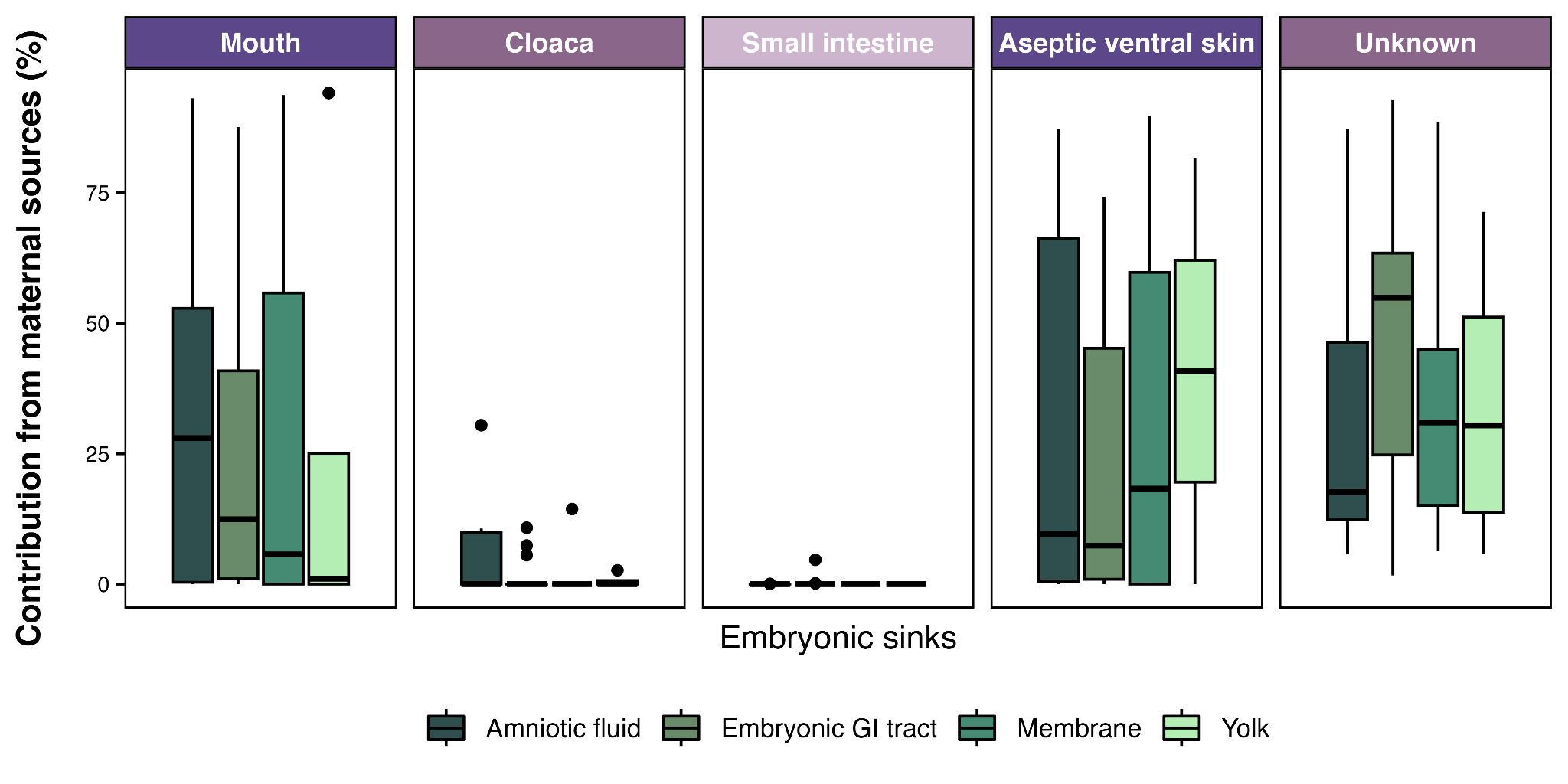


**FIG S2** Source tracking of the bacterial origin of embryos of *Sceloporus grammicus* (Wiegmann, 1828) using the FEAST (Fast Expectation-Maximization for Microbial Source Tracking) algorithm.
